# Use and interpretation of hemoglobin concentrations for assessing anemia status in individuals and populations: results from a WHO technical meeting

**DOI:** 10.1111/nyas.14090

**Published:** 2019-04-21

**Authors:** Maria Nieves Garcia-Casal, Sant-Rayn Pasricha, Andrea J. Sharma, Juan Pablo Peña-Rosas

**Affiliations:** 1 Evidence and Programme Guidance Unit, Department of Nutrition for Health and Development, World Health Organization, Geneva, Switzerland.; 2 Walter and Eliza Hall Institute of Medical Research, Melbourne, Victoria, Australia.; 3 Division of Nutrition, Physical Activity and Obesity, Centers for Disease Control and Prevention, Atlanta, Georgia.; 4 U.S. Public Health Service Commissioned Corps, Atlanta, Georgia

**Keywords:** hemoglobin, thresholds, anemia, public health, clinical significance, technical meeting

## Abstract

Anemia is an important public health problem that negatively affects health of individuals and economic potential of populations. An accurate case definition is critical for understanding burden and epidemiology of anemia, for planning public health interventions, and for clinical investigation and treatment of patients. The current threshold hemoglobin concentrations for diagnosis of anemia were proposed in 1968 and based on studies predominantly of Caucasian adult populations in Europe and North America. The World Health Organization is undertaking a project to review global guidelines for anemia. We describe the process of obtaining input from technical experts, researchers, blood bank experts, policy makers, and program implementers to identify key information or knowledge gaps for anemia diagnosis. From this scoping exercise, six priority areas were identified on diverse topics related to the use and interpretation of hemoglobin concentrations to diagnose anemia in individuals and populations. A call for authors was conducted to produce background, review, and research papers across priority topics. This paper summarizes the first technical meeting, which included commissioned papers as well as case studies, describes key data gaps identified, and describes the next steps in the guideline development process to assess available evidence and define knowledge gaps to improve anemia characterization.

## Introduction

Anemia is a condition in which the number of red blood cells or their oxygen-carrying capacity is insufficient to meet physiologic needs. Definitions of hemoglobin thresholds used to diagnose anemia vary by age, sex, elevation above sea level, smoking, and pregnancy status.^1^ Accurate characterization of anemia is critical to understanding the burden and distribution of this condition, for planning public health interventions, and for comprehensive and integrated health services (promotive, protective, preventive, curative, rehabilitative, and palliative) throughout the life course, prioritizing primary care and essential public health functions. The threshold hemoglobin concentration for diagnosis of anemia, used in the clinical investigation and management of patients, is presently defined below the putative 95% reference range in normal individuals adjusted for age, sex, and pregnancy status, with corrections for elevation of place of residence and smoking practices.^2^ Definitions of anemia have impacts both on public health programs and on clinical decision making.

Hemoglobin thresholds to define anemia were first proposed by the World Health Organization (WHO) in 1959. Current thresholds recommended by WHO for men, women, young children, and during pregnancy were proposed in 1968 after technical meetings with clinical and public health experts working with the evidence available at the time;^3^ data consisted of studies predominantly of Caucasian adult populations in Europe and North America.^4–7^ Data from other countries, ethnic groups, and ages (i.e., infants, young children, adolescents, and the elderly) were not available to the panel.^8^

There are concerns that current thresholds may not appropriately define anemia in all populations to which they have been applied; for example, in individuals of African or Asian populations, in young children and older persons, and in different stages of pregnancy. Hemoglobin thresholds that appropriately indicate a detrimental health outcome or underlying symptoms remain undefined. There are also uncertainties concerning optimal testing methods for hemoglobin concentration.

The prevention and control of anemia is one of the WHO 2025 global nutrition targets.^9^ Improved definitions of anemia would allow a more accurate assessment of the determinants, their burden, and hence the impact of this condition on individuals and in populations.^2,10^ From a public health point of view, governments, nongovernmental organizations, and donors will be able to better target nutrition-specific and -sensitive interventions in primary health care, monitor the effectiveness of programs, and make informed decisions regarding allocation of resources to address the determinants of health, leaving no one behind. From a clinical perspective, measurement of hemoglobin is one of the most commonly performed laboratory tests in the primary health care, hospital, and critical care setting.^11,12^ Updated definitions of anemia would help health professionals manage, investigate, and treat their patients.^13,14^

## WHO project to review hemoglobin thresholds to diagnose anemia

The WHO is undertaking a project to review its global guidelines for hemoglobin thresholds used to define anemia at the individual and population level and determine the impact of health interventions.

As the first step, more than 4000 technical experts, researchers, blood bank experts, policy makers, and program implementers were asked to identify priority questions to understand the key information and knowledge that would enable a revised definition of hemoglobin thresholds, in the form of a prioritized list of scoping questions (http://www.who.int/nutrition/events/2016_online_consultation_haemoglobin_anemia/en/). Over 500 questions from more than 150 respondents were received and consolidated into 58 questions across six categories that were ranked by responders as the most relevant aspects on anemia diagnosis. The six categories were: physiology of anemia, hemoglobin cutoffs for different population groups, definition of anemia across clinical and environmental contexts, approaches to develop anemia cutoffs, laboratory and diagnostic considerations, and the use of WHO hemoglobin cutoffs guidelines. Based on the questions and research needs that scored highest by stakeholders, top ranked questions were identified.^15^

A call for authors was launched to invite clinicians, hematologists, physicians, nutritionists, epidemiologists, health economists, basic scientists, and researchers interested in preparing review papers on the six categories identified through the prioritization exercise and other diverse topics related to the use and interpretation of hemoglobin concentrations to diagnose anemia in individuals and in populations (http://www.who.int/nutrition/callforauthors_anemia_status/en/). The topics requested were: (1) pathophysiology of anemia; (2) effects of genetic variants on hemoglobin concentration; (3) variation in hemoglobin thresholds for anemia across the life cycle; (4) hypoxia, altitude, and other psychobiological aspects affecting hemoglobin concentrations; (5) effect of maternal hemoglobin levels on mother and child health; (6) effect of hemoglobin concentrations on cognitive and physical development in children; (7) defining anemia as a public health problem and classifying severity; (8) hemoglobin for monitoring clinical and nutrition-specific/nutrition-sensitive interventions; (9) optimal methods for hemoglobin measurement in clinical laboratories and field studies; (10) ethics, human rights, and determinants of equity in access to anemia diagnosis; (11) modeling of cutoff points for diagnosing anemia; and (12) country experiences and case studies (Table 1). Thirty-three paper proposals covering 11 of these 12 topics were received, and 17 papers were commissioned after editorial review.

## WHO technical meeting

The Evidence and Programme Guidance Unit, Department of Nutrition for Health and Development of the World Health Organization convened the technical meeting: “Use and interpretation of hemoglobin concentrations for assessing anemia status in individuals and populations,” held in Geneva, Switzerland in November 29–30 and December 1, 2017 (http://www.who.int/nutrition/events/2017-meeting-haemoglobin-concentrations-anemia-29novto1dec/en/).

The objectives of the meeting were to review:

1. The definition of anemia as a public health problem and classification of severity.
2. Variation in hemoglobin thresholds for anemia across the life cycle and other psychobiological aspects affecting hemoglobin concentrations.
3. The effect of hemoglobin levels on maternal and child health, including cognitive and physical development in children.
4. The diagnostic value of hemoglobin concentration for monitoring clinical and nutrition-specific/nutrition-sensitive interventions.
5. Methods for hemoglobin measurement in clinical laboratories and field studies.
6. Ethics, human rights, and determinants of equity in access to anemia diagnosis.
7. Country-level experiences and lessons learned about hemoglobin determinations and anemia diagnosis.
8. Research priorities to fill data gaps related to nutrition interventions to reduce anemia and unintended adverse effects.

The technical meeting was based on, but not limited to, the background papers and case studies that were commissioned through the public call for papers. It included the presentation of the commissioned papers and other topics of interest, plenary discussions, and a session for developing a scoping document as a base for developing PICO questions (Populations, Interventions, Comparisons, Outcomes) that will help with the development of WHO guidelines in the near future.

One of the outcomes of the WHO project on reviewing hemoglobin thresholds is the publication of a special issue in a peer-reviewed journal, containing the commissioned papers and results from this technical meeting. This introductory paper to the special issue provides the background and rationale of the technical consultation, synopsizes the presentations, summarizes the main considerations proposed at the meeting, and identifies the research needs and the way forward for the assessment of anemia.

The meeting opened with presentations high-lighting the importance of anemia as part of the WHO 2025 global nutrition targets,^9^ indicating that anemia is at the heart of the health and development agenda and that there are many aspects that are unclear or unknown about definitions, prevalence, significance, or the proportion of anemia that is responsive to nutritional or other health measures. The objectives of the WHO anemia project and the objectives of the meeting were presented, and participants were invited to challenge the current definitions of anemia and the review of thresholds from a clinical and from a public health point of view, considering implications and consequences of each approach. Participants were reminded that ultimately the aim of the project is to obtain accurate and justifiable definitions of anemia and to define guidelines for its assessment.

There was an introductory group of presentations to set the basis for discussions: definition of anemia and causes and consequences;^10,16^ WHO anemia prevalence estimates;^17–19^ genetic variations associated with hemoglobin across populations; and the biology of autoimmune hemolytic anemia. The first plenary session focused on the high prevalence of anemia worldwide and showed that anemia tends to be most prevalent in areas of the world where nutritional, infectious, and genetic causes of anemia are common. The need to standardize the definition and recognizing the need to include factors affecting the etiology of anemia when establishing hemoglobin thresholds were discussed. Addressing ethnicity in the definition of anemia was proposed, as well as considerations about its role in influencing thresholds. It was observed that evidence for thresholds for infants and older persons was inadequate, that differences in thresholds between the sexes should be considered for all age groups, and that how genetic variation should be accounted for is an unresolved issue that clearly affects some settings more than others. It was emphasized that different settings may require distinct thresholds for anemia given the genetic variations associated with anemia.

There was a group of related presentations that addressed the statistical and functional changes in definitions of anemia over the life course. The paucity of evidence for different thresholds over the life course in different geographic settings was described. The results from a systematic review on associations and effects of increased hemoglobin in preschool children on growth, development, and chronic disease; the role of etiology of anemia and timing of measurement on maternal hemoglobin concentrations and maternal and child health; and the results from another systematic review on the effects of maternal hemoglobin concentrations on maternal and child health in different WHO regions of the world were presented. This group of presentations highlighted concerns related to anemia cutoffs by age, gender, and ethnicity. From the data presented, there was inadequate evidence to tie hemoglobin concentrations to functional outcomes. However, maternal anemia was correlated to negative birth outcomes, but correlations were less clear for maternal outcomes.

The effects of physiologic hypoxia on hemoglobin thresholds were considered next. The need for adjustments on hemoglobin cutoffs for elevation above sea level and smoking was addressed through a systematic review, an analysis of pooled multicountry data, and description of a country experience. In a systematic review of reference values for hemoglobin in populations living at varying altitude, the presenters found evidence that humans may adapt to hypoxia from altitude differently based on ethnicity,^20,21^ mediated via a polymorphism in genes involved in sensing hypoxia. Although there are limited data, at the same altitude Tibetan children have lower hemoglobin concentrations compared to Han Chinese, while Andean children have higher hemoglobin concentrations compared to Han; these findings may be explained by genetic differences in populations.^22^ The other analysis used amalgamated data from 14 population-based nutrition surveys among 13 countries from the Biomarkers Reflecting Inflammation and Nutritional Determinants of Anemia (BRINDA) project,^23,24^ and studies containing data on altitude, smoking, and hemoglobin (Bolivia and Guatemala) to examine effects of elevation above sea level and smoking on hemoglobin concentration. The experience from Bolivia in children under 5 years of age included a comparative analysis among three regions of different altitudes showing a high prevalence of anemia, and that the factors associated with anemia in children varied by elevation above sea level. Smoking, electronic cigarettes, indoor cooking stoves, and effects on hemoglobin were discussed. Collectively, these studies indicated that current hemoglobin adjustments for altitude may be too large, especially at higher altitudes, and may require reconsideration.

The next block of presentations was related to the definitions of severity of anemia at the clinical level, and the definition of mild, moderate, and severe burdens of anemia at the public health level. It was clear that an evidence-based definition of hemoglobin thresholds is needed for clinical thresholds. The causes of anemia, especially the contribution of iron deficiency to anemia severity, were constantly addressed during discussions. The role of hemoglobinopathies, infections, and inflammation—of any origin and degree—needs to be taken into consideration when defining hemoglobin cutoff points. The evidence for transfusion thresholds was also addressed, highlighting that the need and point for initiating transfusion deserves careful consideration and clear criteria for donors and transfused individuals.^25–27^

Laboratory methodologies for assessment of hemoglobin concentrations were discussed. After three presentations, participants discussed concerns about reproducibility of methods and feasibility in field studies. Concern was expressed about comparability and performance between different platforms, especially the HemoCue® system compared with others and between different editions of the same instrument/system. A key emerging problem observed in field epidemiologic studies is the divergence in venous and capillary hemoglobin measurements; these discrepancies raise the possibility that current estimates of the global prevalence of anemia are overestimated as most population-based estimates used capillary measures of hemoglobin. Concern was expressed about the HemoCue system having proprietary quality controls, the frequency of routine use of these controls in field practice, and the need to ensure calibration materials for all instruments are commutable to international reference materials/standards. The practicality of implementing various assays in field studies, techniques for laboratory quality control, strategies for field workers, and phlebotomist training were also discussed. The need for guidance on controlling for these various critical preanalytic aspects was emphasized.

Finally, the meeting addressed other factors affecting anemia prevalence. There were presentations on the complex interaction between malaria, iron metabolism, and anemia; associations between water sanitation and hygiene conditions and anemia prevalence; and the role of nutrition-related interventions for preventing and controlling anemia through the life cycle. Ethical and human rights considerations related to the accuracy and interpretation of hemoglobin concentrations for anemia diagnosis were also explored.

## Closing remarks and research needs

The final session of the meeting was devoted to in-depth discussions and clarifications, and to launching an approach to defining PICO questions, which are critical to scoping future WHO guidelines and identifying knowledge gaps, and hence research priorities critical to developing the evidence needed to support the guidelines. Other aspects, such as anemia severity, public health significance, and laboratory methods, were also considered.

The meeting highlighted key uncertainties in incumbent thresholds and approaches to defining anemia. Importantly, there is negligible evidence to support current hemoglobin thresholds in infancy, childhood, pregnancy, and the elderly. Likewise, it is unclear whether the same thresholds should be used across different ethnicities, or how genetic propensity to hemoglobin variants should be accounted for in individual clinical diagnosis and population estimates of anemia prevalence. The hemoglobin concentrations associated with acute clinical adverse events (i.e., symptomatic anemia or exacerbation of medical conditions in individuals with chronic diseases, such as ischemic heart disease) are uncertain. The hemoglobin thresholds below which adverse functional outcomes are observed in children (e.g., impaired future development) and pregnancy (e.g., diminished birth weight) are likewise uncertain. Uncertainty in anemia definitions is reflected by heterogeneity in thresholds recommended by professional organizations.

Several crucial questions must be considered to improve current hemoglobin thresholds to define anemia. Diagnostic thresholds can be defined through a variety of approaches. First, anemia could be defined by hemoglobin levels below the reference range, that is, below a statistical centile (e.g., the 2.5th centile of hemoglobin in a healthy population). Alternatively, anemia may be considered to reflect thresholds below which clinical symptoms (e.g., fatigue and lethargy) or exacerbation of underlying clinical conditions (e.g., congestive cardiac failure or ischemic heart disease) appear. Anemia could also be defined as hemoglobin levels below which individuals are at risk of functional adverse consequences—for example, in pregnant women, reduced birth weight, or prematurity; and in infants, reduced cognitive development. Finally, it may be critical to detect anemia at a hemoglobin threshold where individuals have a risk of an underlying clinical or medical condition that necessitates further investigation—for example, a genetic condition (e.g., carriage of thalassemia), a nutritional deficiency, inflammation, or chronic bleeding.

It will be important to consider groups of individuals for whom thresholds may need to vary—for example, thresholds currently differ between under-5 children, school-age children, and adults; between adult males and females; and between nonpregnant and pregnant adult females.^8^ It is important to consider whether these groups are appropriate or whether more narrowly defined group-specific thresholds are required—for example, among younger infants, across different trimesters of pregnancy, and in the elderly. Likewise, it will be important to consider whether the same thresholds are appropriate in all populations and across different countries and in people of different ethnicities, or whether different thresholds should be developed.

There remains considerable uncertainty regarding preanalytic variables associated with hemoglobin measurement, especially the differences in hemoglobin concentration between venous and capillary blood collection, and whether these can be sufficiently overcome for routine use of capillary measurement in the field. There is also concern regarding the validity of hemoglobin measurements using point-of-care analyzers compared with automated hematology analyzers. In low- and middle-income countries, other techniques for hemoglobin estimation are still used, for example, filter paper methods, manual cyanmethemoglobin measurement using spectrophotometry, and other measures. Noninvasive techniques for assessment of hemoglobin measurement are entering clinical practice in some settings and may become more established in the medium term. However, the validity of these methods needs to be determined.

At the population level, it will be important to consider how the public health relevance of anemia prevalence should be determined. This is critical to determine settings in which nutrition-specific (e.g., iron supplementation) or-sensitive (e.g., malaria control) interventions may be beneficial. Currently, the population-level problem of anemia is predominantly derived from estimates in under-5 children and women; are these the optimal and only groups that should be considered in routine surveys and global estimates?

## Guideline development

WHO will convene a formal Guideline Development Process to assess the available evidence and define critical evidence needs. Some aspects may be able to be addressed using available evidence—for example, analyses of effects of altitude on thresholds. However, for other issues, new data and evidence will be needed.

Based on what was presented at the Consultation, there are minimal data to support hemoglobin thresholds to define anemia. There is a paucity of evidence for thresholds across the life cycle and in individuals of different ethnicities and from different countries. A multicenter hemoglobin reference study (akin to similar international studies such as the WHO multicenter growth reference study and the WHO fetal growth study) could define the 2.5th centile across healthy individuals of different age groups and geographic settings. Such a study would confirm whether thresholds are consistent across different populations and provide an opportunity to confirm the effects of genetic variants (e.g., thalassemia carriage) on thresholds. The need and possible design for such a study is presently being defined.

While anemia is associated with adverse outcomes in pregnancy, evidence linking anemia to adverse developmental outcomes in infants is less clear. In both cases, evidence for specific thresholds that link to functional outcomes is absent. Individual patient data meta-analysis of cohort studies, or analysis of prospective cohort data, could define these associations and identify hemoglobin thresholds correlated to functional detriments.

Detailed studies to compare hemoglobin levels assessed across different preanalytic specimens and using different analytic platforms are necessary to enable interpretation of data from surveys, and to confirm validity of studies and clinical testing using these different approaches. Additionally, considerations of including methodological recommendations about sampling, source of blood, storage conditions and duration, volumes, training, international standards, calibration materials, and analytical systems—automated hematology analyzers, point-of-care analyzers, cyanmethemoglobin measurement by spectrophotometry, filter paper-based methods, clinical evaluation (e.g., examination for conjunctival or palmar pallor), or noninvasive measures (i.e., pulse oximetry)—in guidelines should be addressed.

Despite the limitations of available data, the complex questions raised, and the clear need for information, outcomes of this technical meeting contribute to the Member States’ efforts to strengthen their health systems. This supplement provides a summary of technical considerations that can be useful in developing policies around diagnosis of anemia and its causes to inform implementation of nutrition-specific and nutrition-sensitive programs. It will ultimately contribute to WHO’s ongoing normative work in this field.

## Figures and Tables

**Table 1.** Topics and objectives of papers requested through the WHO call for authors

Topic	Objectives
1. Pathophysiology of anemia	To provide an overview of etiology and pathophysiology of anemias; classification of anemia into its constituent causes and mechanisms by which each cause may produce anemia; deficiencies or aberrant metabolism of hematinic micronutrients (iron, B12, and folate) and other possible micronutrients (e.g., vitamin A, vitamin D, and riboflavin) as causes of anemia. Mechanisms and epidemiology of anemia of inflammation. This paper would be expected to incorporate both mechanistic data from experimental studies and population health data. The author would be requested to provide some input into how each cause of anemia may influence hemoglobin thresholds and whether these have implications for the definition of anemia.
2. Effects of genetic variants on hemoglobin concentration	To summarize the effect of different genetic variants and ethnicities on hemoglobin concentration. This would be expected to encompass variants in hemoglobin (thalassemias and hemoglobinopathies), red cell enzyme disorders, and red cell membrane disorders. Include considerations on the role of genetics in overall variation in hemoglobin concentrations and the potential impact ofgenetics on definitions of hemoglobin thresholds used to define anemia. Considerations on the effects of clinically silent, “minor” or carrier hemoglobin phenotypes and consideration ofSNPs in genes regulating iron status (e.g., TMPRSS6) would be important.
3. Variation in hemoglobin thresholds for anemia across the life cycle	To summarize evidence for possible variation in hemoglobin thresholds; to define anemia between males and females, and across key stages of the life cycle: neonates, children < 1 year of age, preschool children, primary school-aged children, adolescents, and adults, including premenopausal women, postmenopausal women, and the elderly.
4. Hypoxia, altitude, and other psychobiological aspects affecting hemoglobin concentrations	Summary of current evidence for how hemoglobin concentrations are affected by altitude. Discussion of the effects and mechanisms by which hypoxia influences erythropoiesis. Evaluation of current WHO approaches to adjusting hemoglobin thresholds. Effect of smoking; use of electronic cigarettes.
5. Effect of maternal hemoglobin levels on mother and child health	Summary of the evidence for the association between maternal hemoglobin concentrations and maternal and infant outcomes; evaluating associations between hemoglobin levels in a continuous manner, rather than just anemia per se. Outcomes of interest include, but are not limited to, maternal mortality, hospitalization, infection, hemorrhage, transfusion, antepartum and postpartum well-being, postpartum depression; infant birth weight, birth length, gestational age, weight for gestational age, child growth, child development, and long-term health outcomes. Any evidence for thresholds of hemoglobin that predict risk (or protection) from these outcomes should be emphasized.
6. Effect of hemoglobin concentrations on cognitive and physical development in children	Summary of the evidence for the association between child hemoglobin concentrations and long-term growth and developmental outcomes; evaluating associations between hemoglobin levels in a continuous manner, rather than just anemia per se. Outcomes of interest include, but are not limited, to child longitudinal and ponderal growth, child cognitive, psychomotor, and behavioral development, long-term health outcomes, including the risk of chronic disease. Any evidence for thresholds of hemoglobin that predict risk (or protection) from these outcomes should be emphasized.
7. Defining anemia as a public health problem and classifying severity	To define how the severity of the burden of anemia should be considered at a population level. Should this be defined in terms of prevalence of anemia, disability-adjusted life years, or economic costs?A health economic approach is expected to be incorporated into this analysis. Discussion of the possible effect of revised definitions ofhemoglobin thresholds should be included. To conceptualize how different anemia severities may be defined at the individual (clinical) and public health level—exclusively based on Hb concentrations, based on clinical symptoms or signs, on need for treatments (e.g., transfusion and iron supplementation). To discuss how a decision to treat anemia should be made at both the clinical and population health level. To provide a proposed research framework for defining mild/moderate/severe anemia in individuals.
8. Hemoglobin for monitoring clinical and nutrition- specific/nutrition-sensitive interventions	To provide evidence that effects from interventions designed to correct anemia levels either directly or indirectly can be measured using hemoglobin. For example, do interventions, such as transfusion, iron infusions, or supplementation, other micronutrient interventions, for example, B12, folate, or vitamin A, raise hemoglobin? Is hemoglobin a reliable biomarker of response to these interventions? Likewise, do indirect (e.g., nutrition-sensitive interventions, correction of inflammation, treatment ofinfection, malaria control, or water and sanitation) measures raise hemoglobin? Regarding iron interventions, what is the proportion of anemia presently considered attributable to iron deficiency in global health? What proportion of the burden of anemia may be expected to respond to iron?
9. Optimal methods for hemoglobin measurement in clinical laboratories and field studies	To summarize the range ofmethodologies used to measure hemoglobin concentrations in (1) clinical laboratories and (2) field studies. It is important to provide some description of the principles of each assay. The authors should discuss benefits and limitations of each method. Currently considered best practice for use in clinical and field studies should be discussed in detail. In addition, authors are requested to provide some input into quality control aspects of laboratory and field measurements. To provide a summary of the evaluation of anemia from a clinical and public health perspective. What other clinical information and tests should be undertaken at the same time as measuring hemoglobin in order to provide insight into the underlying cause of the anemia and its severity. What other biomarkers should be measured along with hemoglobin to assess the cause/severity of anemia in public health surveys and studies? What would be the costs (financial, and in terms of anxiety and false positive/negative diagnoses) if these tests were concurrently ordered? What are the practical implications (e.g., costs, sample volumes, appropriate collection tubes, and access to testing) if these additional tests were requested?
10.Ethics, human rights, and determinants of equity in access to anemia diagnosis	Anemia diagnosis as a human right. Physiological differences and technical aspects affecting access and equity aspects to anemia diagnosis, including differential classification as a mild, moderate, or severe magnitude of a public health problem, gender, geographical location, and accessibility. To identify factors preventing access to anemia diagnosis (not only hemoglobin determinations across social groups (e.g., women, children, elderly, and rural populations)), especially among those who are most vulnerable to anemia. Micronutrient deficiencies. Ethical considerations when performing population studies involving vulnerable groups.
11.Modeling of cutoff points for diagnosing anemia	Blood banks, biobanks, and other academic, clinical, or educational databases in different countries with “healthy” individuals and the feasibility for developing cutoffpoints to diagnose anemia. Proposals based on evidence and mathematical models on cutoff points for anemia for all gender, age, physiological and pathophysiological conditions, cultural, geographical, or traditions groups where there is not enough evidence to propose a cutoffpoint.
12.Country experiences and case studies	To describe the country’s experience and history with anemia diagnoses based on hemoglobin determination and the validation of other markers at population level. Description of successful programs on changing prevalence of anemia and/or those without changes in prevalence of anemia, but improved hemoglobin concentrations.

Note: Topics were selected from the six categories identified by the prioritization exercise.

## Statement

This manuscript summarizes the presentations and discussions from the World Health Organization (WHO) technical consultation “Use and interpretation of hemoglobin concentrations for assessing anemia status in individuals and populations,” held in Geneva, Switzerland in November 29–30 and December 1, 2017. This paper is being published individually but will be consolidated with other manuscripts as a special issue of *Annals of the New York Academy of Sciences*, the coordinators of which were Maria Nieves Garcia-Casal and Sant-Rayn Pasricha. The special issue is the responsibility of the editorial staff of *Annals of the New York Academy of Sciences*, who delegated to the coordinators preliminary supervision of both technical conformity to the publishing requirements of *Annals of the New York Academy of Sciences* and general oversight of the scientific merit of each article. The workshop was supported by WHO, Centers for Disease Control and Prevention (CDC), USA; the United States Agency for International Development (USAID), USA; and the Bill & Melinda Gates Foundation, USA. The authors alone are responsible for the views expressed in this paper; they do not necessarily represent the views, decisions, or policies of the WHO or the Centers for Disease Control and Prevention. The findings and conclusions in this report are those of the authors and are not attributable to or represent the official position of the World Health Organization, the Centers for Disease Control and Prevention, the sponsors, publisher, or editorial staff of *Annals of the New York Academy of Sciences*.

## References

[1] World Health Organization. 2014 Global nutrition targets 2025: anemia policy brief (WHO/NMH/NHD/14.4) Geneva: World Health Organization.

[2] World Health Organization. 2001 Iron deficiency anemia: assessment, prevention and control. A guide for programme managers Geneva: World Health Organization.

[3] World Health Organization. 1968 Nutritional anemias: report of a WHO Scientific Group 1968. Report No.: 405 Geneva: World Health Organization. 4975372

[4] Sturgeon P 1959 Studies of iron requirements in infants. III. Influence of supplemental iron during normal pregnancy on mother and infant A. The mother. Br. J. Haematol 5: 31–44. 13628927 10.1111/j.1365-2141.1959.tb04011.x

[5] Kilpatrick GS & Hardisty RM. 1961 The prevalence of anemia in the community: a survey of a random sample of the population. Br. Med. J 1: 778–782. 13830948 10.1136/bmj.1.5228.778 PMC1953914

[6] de Leeuw NKM, Lowenstein L & Hsieh YS. 1966 Iron deficiency and hydremia in normal pregnancy. Medicine 45: 291–315. 5946689 10.1097/00005792-196607000-00002

[7] Natvig K 1966 Studies on hemoglobin values in Norway: V. Hemoglobin concentration and hematocrit in men aged 15–21 years. Acta Med. Scand 180: 613–620. 5923383

[8] World Health Organization. 2011 Hemoglobin concentrations for the diagnosis of anemia and assessment of severity. Vitamin and Mineral Nutrition Information System. (WHO/NMH/NHD/MNM/11.1) Geneva: WHO Accessed August 3, 2018 http://www.who.int/vmnis/indicators/haemoglobin.pdf.

[9] World Health Organization. 2014 Global nutrition targets 2025: policy brief series (WHO/NMH/NHD/14.2) Geneva: World Health Organization.

[10] World Health Organization. 2017 Nutritional anemias: tools for effective prevention and control Geneva: World Health Organization.

[11] Broderick AJ 2015 Point of care hemoglobin measurement—state of the art or a bleeding nuisance? Anaesthesia 70: 1225–1229. 26374281 10.1111/anae.13231

[12] Hoff CM, Hansen HS, Overgaard M, 2011 The importance of hemoglobin level and effect of transfusion in HNSCC patients treated with radiotherapy—results from the randomized DAHANCA 5 study. Radiother. Oncol 98: 28–33. 20970213 10.1016/j.radonc.2010.09.024

[13] Barker SJ, Shander A & Ramsay MA. 2016 Continuous noninvasive hemoglobin monitoring: a measured response to a critical review. Anesth. Analg 122: 565–572. 25746056 10.1213/ANE.0000000000000605 PMC4708068

[14] Hare G, Tsui A, Ozawa S & Shander A. 2013 Anemia: can we define hemoglobin thresholds for impaired oxygen homeostasis and suggest new strategies for treatment? Best Pract. Res. Clin. Anaesthesiol 27: 85–98. 23590918 10.1016/j.bpa.2012.12.002

[15] Pasricha SR, Colman K, Centeno-Tablante E, 2018 Revisiting WHO hemoglobin thresholds to define anemia in clinical medicine and public health. Lancet Haematol 5: e60–e62. 29406148 10.1016/S2352-3026(18)30004-8

[16] Beutler E & Waalen J. 2006 The definition of anemia: what is the lower limit of normal of the blood hemoglobin concentration? Blood 107: 1747–1750. 16189263 10.1182/blood-2005-07-3046 PMC1895695

[17] Stevens GA, Finucane MM, De-Regil LM, 2013 Global, regional, and national trends in hemoglobin concentration and prevalence of total and severe anemia in children and pregnant and non-pregnant women for 1995–2011: a systematic analysis of population-representative data. Lancet Glob. Health 1: e16–e25. 25103581 10.1016/S2214-109X(13)70001-9 PMC4547326

[18] World Health Organization. 2008 Worldwide prevalence of anemia 1993–2005 WHO Global Database on Anemia Geneva: World Health Organization.

[19] World Health Organization. 2015 The global prevalence of anemia in 2011 Geneva: World Health Organization.

[20] Gassmann M & Muckenthaler M. 2015 Adaptation of iron requirement to hypoxic conditions at high altitude. J. Appl. Physiol 119: 1432–1440. 26183475 10.1152/japplphysiol.00248.2015

[21] Haase V 2013 Regulation of erythropoiesis by hypoxiainducible factors. Blood Rev 27: 41–53. 23291219 10.1016/j.blre.2012.12.003 PMC3731139

[22] Simonson TS, Yang Y, Huff CD, 2010 Genetic evidence for high-altitude adaptation in Tibet. Science 329: 72–75. 20466884 10.1126/science.1189406

[23] Engle-Stone R, Grant A, Huang J, 2017 Predictors of anemia in preschool children: Biomarkers Reflecting Inflammation and Nutritional Determinants of Anemia (BRINDA) Project. Am. J. Clin. Nutr 106: 402S–415S. 28615260 10.3945/ajcn.116.142323 PMC5490650

[24] Stoltzfus R & Klemm R. 2017 Research, policy, and programmatic considerations from the Biomarkers Reflecting Inflammation and Nutritional Determinants of Anemia (BRINDA) Project. Am. J. Clin. Nutr 106: 428S–434S. 28615252 10.3945/ajcn.116.142372 PMC5490649

[25] World Health Organization. 2001 Developing a national policy and guidelines on the clinical use of blood. Guidelines. Geneva: World Health Organization.

[26] World Health Organization. 2016 Establishing external quality assessment programmes for screening of donated blood for transfusion-transmissible infections: implementation guide Geneva: World Health Organization.

[27] World Health Organization. 2016 A guide to establishing a national haemovigilance system Geneva: World Health Organization.

